# Ocean forcing drives glacier retreat in Greenland

**DOI:** 10.1126/sciadv.aba7282

**Published:** 2021-01-01

**Authors:** Michael Wood, Eric Rignot, Ian Fenty, Lu An, Anders Bjørk, Michiel van den Broeke, Cilan Cai, Emily Kane, Dimitris Menemenlis, Romain Millan, Mathieu Morlighem, Jeremie Mouginot, Brice Noël, Bernd Scheuchl, Isabella Velicogna, Josh K. Willis, Hong Zhang

**Affiliations:** 1 Department of Earth System Science, University of California Irvine, Irvine, CA 92697, USA.; 2 Jet Propulsion Laboratory, California Institute of Technology, Pasadena, CA 91109, USA.; 3 Department of Geosciences and Natural Resource Management, University of Copenhagen, Copenhagen, Denmark.; 4 Institute for Marine and Atmospheric Research, Utrecht University, Utrecht, Netherlands.; 5 University of Grenoble Alpes, CNRS, IRD, Grenoble INP, IGE, Grenoble, France.

## Abstract

The retreat and acceleration of Greenland glaciers since the mid-1990s have been attributed to the enhanced intrusion of warm Atlantic Waters (AW) into fjords, but this assertion has not been quantitatively tested on a Greenland-wide basis or included in models. Here, we investigate how AW influenced retreat at 226 marine-terminating glaciers using ocean modeling, remote sensing, and in situ observations. We identify 74 glaciers in deep fjords with AW controlling 49% of the mass loss that retreated when warming increased undercutting by 48%. Conversely, 27 glaciers calving on shallow ridges and 24 in cold, shallow waters retreated little, contributing 15% of the loss, while 10 glaciers retreated substantially following the collapse of several ice shelves. The retreat mechanisms remain undiagnosed at 87 glaciers without ocean and bathymetry data, which controlled 19% of the loss. Ice sheet projections that exclude ocean-induced undercutting may underestimate mass loss by at least a factor of 2.

## Teaser

Many Greenland glaciers have been attacked by the ocean from below and, in turn, contributed to sea-level rise from the ice sheet.

## INTRODUCTION

The Greenland Ice Sheet has contributed substantially to sea-level rise over the past few decades. Since 1972, approximately two-thirds of the ice sheet’s contribution to sea-level rise resulted from increased glacier flux with the remaining one-third from anomalous surface melt (*1*). Before 2000, anomalous ice discharge was the dominant driver of mass loss, but in recent years, increasingly negative surface mass balance anomalies have contributed to a larger proportion of the total mass loss from the ice sheet (*1*). The acceleration in mass flux has been partially attributed to a warming of subsurface waters around Greenland near the end of the 1990s (*2*, *3*) and increased runoff, resulting in enhanced water mixing and melt at glacier margins, destabilization of terminus regions (*4*, *5*), ice front retreat (*6*, *7*), and, in most cases, accelerated ice flow (*8*). The increase in flow speed, combined with enhanced surface melt, results in increased glacier thinning, which is conducive to further retreat (*9*). Other processes may have additionally contributed to the glacier retreat, e.g., increases in basal lubrication, melting of the ice mélange in front of glaciers, or weakening of glacier shear margins, but quantitative evidence about their impact has been limited (*10*–*12*).

**Fig. 1:**
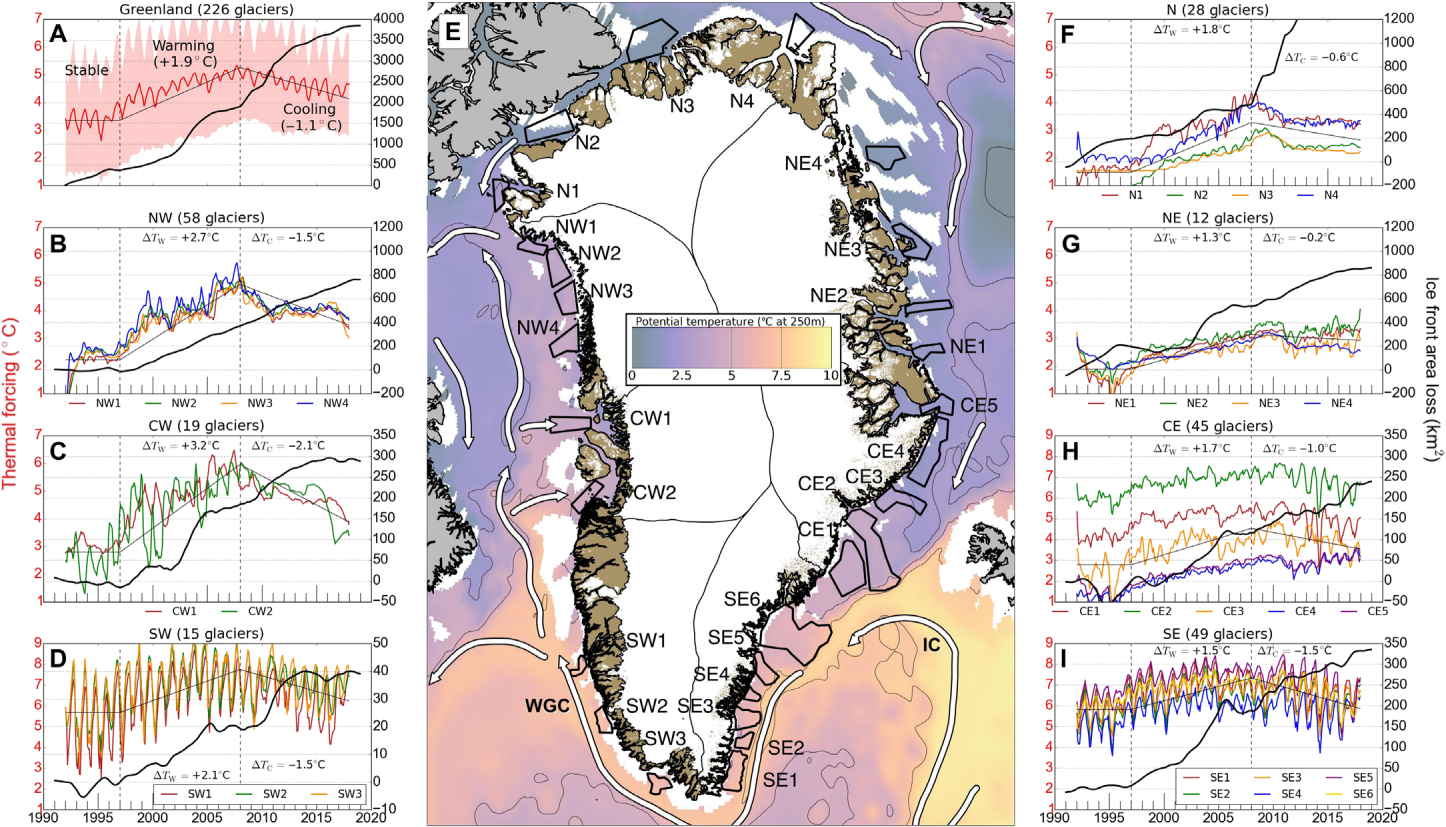
Regional comparison of ocean TF and glacier retreat during 1992–2017. The reconstruction of ocean TF (°C)—the depth-averaged difference between the in situ water temperature and the salt- and pressure-dependent freezing point of seawater—and cumulative glacier retreat (${\hat{Q}}_{r}$, square kilometer) is shown for (**A**) all 226 marine-terminating glaciers (red; ±1σ of all regions), respectively, (**B**) northwest (NW), (**C**) central west (CW), (**D**) southwest (SW), (**F**) north (N), (**G**) northeast (NE), (**H**) central east (CE), and (**I**) southeast (SE) Greenland. Linear regressions in TF through stable, warming, and cooling periods are identified as three thin black lines. (**E**) Sample areas (thick black, numbered by region) used to evaluate TF in seven regions, with major ocean currents (white), overlaid on a reconstruction of potential temperature at 257-m depth from the MITgcm ocean model for November 2005 (*3*). IC, irminger current; WGC, west greenland current.

The warming of subsurface waters at the turn of the 21st century was caused by the spreading of ocean heat from the subpolar gyre during a transition in the North Atlantic Oscillation (NAO) from a high positive phase to a low-to-negative phase (*3*). In this shift, the North Atlantic subpolar gyre expanded, enhancing ocean heat fluxes through the coastal Irminger and West Greenland currents, yielding warmer subsurface waters on the continental shelf of all seven major basins of Greenland (Fig. 1). Since 2010, the NAO has transitioned back to a more positive phase, yielding a relative cooling of the ocean waters, however, not sufficiently to bring back ocean heat fluxes to the levels of the 1990s (*13*).

Estimates of ocean-induced melt from numerical models and in situ data indicate an average melt intensity for the submerged part of the glaciers comparable to, yet generally smaller than, the mean rate of ice advection (*14*). This lower magnitude of melt relative to advection has called into question the role of ocean thermal forcing (TF) on glacier evolution (*15*). However, several studies have noted a coincidence in time between glacial retreat and warmer ocean waters (*2*, *4*) and found a positive correlation between ocean temperature and terminus position (*13*, *16*). This positive correlation has been exploited to linearly approximate the future retreat of tidewater glaciers in response to regional ocean warming (*17*). Yet, there has been no attempt to quantify the spatial and temporal linkages between ice front retreat, dynamic mass loss, and ocean temperature in Greenland fjords on an ice sheet–wide scale. Such assessment has been hindered primarily by a paucity of ocean temperature and bathymetry data in Greenland fjords.

In 2015, NASA launched the Earth Venture Suborbital mission Oceans Melting Greenland (OMG) to begin annual measurements of ocean temperature and salinity with ship-based and airborne expendable conductivity-temperature-depth (CTD) sensors (2015–2019), coastal bathymetry in fjords and on the continental shelf from ship-based multibeam echo sounding (2015–2019) and airborne gravity (2016), and glacier thinning from airborne interferometric surface topographic surveys (2016–2019) (*18*). The new bathymetric measurements were combined with a reconstruction of bed topography beneath the ice sheet to provide a seamless transition in elevation from the seafloor in the fjords to the glacier bed (*19*). This reconstruction, BedMachineV3, provides substantial improvements in estimates of bedrock geometry beneath glaciers and within their fjords, revealing connections between subglacial valleys and submarine troughs and the presence or absence of ridges in the glacial fjords. In addition, CTD data collected in the fjords diagnose the presence (or absence) of subsurface Atlantic Water (AW) in deep (or shallow) fjords and on the continental shelf (*18*). Here, we combine these extensive new observations with ocean modeling to assess the extent to which ocean warming has induced ice front retreat at 226 marine-terminating glaciers that controlled 96% of ice discharge from the Greenland Ice Sheet between 1992 and 2017.

## RESULTS

### Glacier retreat and ocean TF

Between 1992 and 2017, Greenland’s marine-terminating glaciers lost 3536 Gt (gigaton = 10^12^ kg) of mass and 2452 km^2^ of grounded ice (Table 1). The largest retreats occurred in northwest (NW) (28%), north (N) (22%), northeast (NE) (15%), and southeast (SE) (14%) (table S1). To compare grounded ice retreat with oceanic TF, we reconstruct ocean temperature using two ocean state estimates from the Estimating the Circulation and Climate of the Ocean (ECCO) consortium combined with the CTD data (see Materials and Methods). TF is calculated as the depth-integrated difference between the in situ ocean temperature and the salinity- and pressure-dependent freezing point of seawater from the lower 60% of the water column. We identify three periods based on the temporal trends in TF: (i) a stable TF period in 1992–1997, (ii) a warming period of rapid change in 1998–2007 averaging 0.19°C/year, and (iii) a cooling period of slower change in 2008–2017 averaging −0.11°C/year (Fig. 1).

**Table 1:** Grounded ice retreat, ice discharge, and mean mass balance for six glacier categories and three time periods: 1992–1997, 1998–2007, and 2008–2017. The first four categories pertain to glaciers terminating in DW, on CR, in SC (***<***100-m depth), and with FE. The final two categories pertain to glaciers already in an SR, and NC glaciers with no ocean and bathymetry data.

**Category**	**No. of glaciers**	**Ice loss (km^2^)**			**Discharge**	**Mass balance**
		**1992–1997**	**1998–2007**	**2008–2017**	**1992–2017 (Gt/year)**	**1992–2017 (Gt/year)**
DW	74	13.5	601.9	612.9	225.8	−66.3
CR	27	10.0	29.3	32.0	84.0	−12.9
SC	24	3.8	34.8	34.6	20.9	−6.7
FE	10	107.2	192.5	256.7	80.9	−20.6
SR	4	26.3	37.9	57.8	3.2	−3.4
NC	87	19.0	181.8	200.1	62.1	−26.2
Total	226	179.7	1078.2	1194.2	476.9	−136.0

During the stable period 1992–1997, grounded ice retreated 180 km^2^, or 30 km^2^/year, with the largest loss from 10 glaciers terminating in long (>10 km), perennial floating ice shelves (107 km^2^), while four glaciers already in a state of retreat (SR) contributed another 26 km^2^: Kofoed-Hansen Bræ, Academy, Kjer, and the southernmost branch of Upernavik Isstrøm. During the warming period 1998–2007, TF increased by +1.5° to 2°C in SE, central east (CE), and N; +2° to 3°C in southwest (SW) and NW; and more than +3°C in central west (CW) (Fig. 1). Grounded ice loss tripled to 108 km^2^/year. NW accounted for most of the loss (327 km^2^), followed by N (229 km^2^) and SE (163 km^2^). Many ice shelves broke up during this period in front of Jakobshavn Isbræ in CW, Zachariæ Isstrøm in NE, C. H. Ostenfeld and Hagen Bræ in N, and Alison in NW. During the cooling period 2008–2017, TF decreased by 0° to 1°C in CE, NE, and N; 1.5°C in SE, SW, and NW; and 2.1°C in CW, but grounded ice continued to decline at 119 km^2^/year or 14% higher than the previous period. Significant changes persisted on glaciers with ice shelves, with grounded ice retreating at 26 km^2^/year. Petermann and Steensby, in particular, lost large portions of their ice shelves during this period.

### Balance of fluxes at the glacier fronts

To quantify the role of the ocean in controlling the evolution of these glaciers, we calculate the balance of fluxes at the grounded ice fronts that includes (i) grounded ice removed by the ocean (*q*_m_), (ii) ice front retreat caused by glacier thinning (*q*_s_), (iii) ice advection (*q*_f_), and (iv) residual calving of grounded ice blocks (*q*_c_) required to match (v) the observed ice front retreat (*q*_r_) (Materials and Methods). Conservation of mass dictates that *q*_r_ balances ablation from *q*_m_, *q*_s_, and *q*_c_, with advection from *q*_f_.

**Fig. 2:**
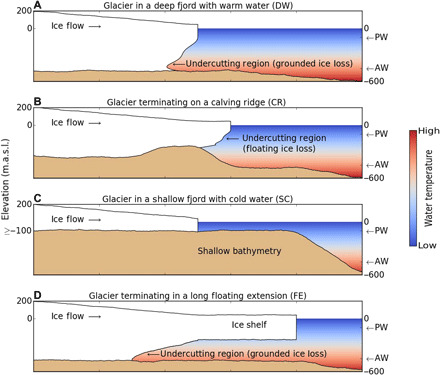
Schematic diagrams for four major categories of marine-terminating glaciers, with cold, fresh polar water (PW) on top of warm, salty AW. (**A**) Glaciers in deep fjords with warm AW (DW) that undercuts the glacier face to affect basal resistance. (**B**) Glaciers with temporary floating extensions on a shallow ridge (SC), for which undercutting does not affect basal resistance. (**C**) Glaciers standing in shallow, cold waters (SC). (**D**) Glaciers develop long (>10 km) floating ice extensions (FE). Note that glacier and bed elevations, expressed in meters above sea level (m.a.s.l.), are approximations provided for illustration.

To reconstruct ice removal by the ocean, we use a model parameterization that quantifies the maximal ice front melt, *q*_m_, on the glacier face at depth (Materials and Methods) (*20*). Model studies using the Massachusetts Institute of Technology (MIT) General Circulation Model (MITgcm) ocean model (*20*, *21*) and in situ scanning of submerged ice faces by multibeam echo sounders (*22*–*24*) have shown that Greenland glaciers do not melt uniformly along their submerged calving margins but melt preferentially at depth, near the glacier base, where ocean temperature is higher, water pressure depresses the freezing point of seawater, and subglacial water generates a buoyant plume of melt water that rises along the submerged glacier face and entrains ocean heat, especially in the summer months. Ocean models show that the maximal melt rate occurs right above (<50 m) the seafloor (*21*, *25*), forming an undercut cavity that reduces basal resistance to glacier flow—ice above the melted incision is not supported from below, while ice under the incision is too thin to resist ice flow from upstream (Fig. 2). From this viewpoint, it is the maximal melt rate at depth (hereafter referred to as the undercutting rate) that has the greatest effect on the glacier force balance (*26*), not the average melt rate of the submerged ice face, which is typically two to three times lower according to the ocean model and does not affect basal friction. In effect, enhanced undercutting forces grounding line retreat. We evaluate *q*_m_ using measurements of water depth, subglacial water discharge, and a reconstruction of TF. Because the resolution of the ECCO state estimates is not sufficient to resolve heat transport from the shelf into the fjords, we use CTDs on the shelf and within the fjords to quantify the modulation of TF between the ocean model calibration areas (Fig. 1) and the glacier fronts (Materials and Methods).

To quantify the anomaly in undercutting, we use a reference rate, $q_{m}^{\text{ref}}$, for the period 1992–1997 when most glaciers were in a state of mass balance and with stable ice fronts (*1*). We calculate the cumulative anomaly in undercutting, ${\hat{Q}}_{m}$, for each glacier as in (*5*). We calculate the thinning-induced retreat, *q*_s_, and its cumulative anomaly, ${\hat{Q}}_{s}$, from observations of ice surface elevation in digital elevation models (DEMs) from 1978 to 1987 (Korsgaard DEM) (*27*), 2002 to 2009 (Greenland Ice Mapping Project DEM) (*28*), and 2015 to 2016 (Arctic DEM) (*29*), combined with airborne altimetry and ice velocity as in (*1*) (see Materials and Methods) ($q_{s}^{\text{ref}}$ is 0). We assume that glaciers retreat when ice fronts have thinned enough to reach floatation. We use remote sensing data to quantify the rate of ice advection, *q*_f_, and the cumulative anomaly, ${\hat{Q}}_{f}$, using a reference, $q_{f}^{\text{ref}}$, for 1992–1997 (see Materials and Methods). For ice front retreat, we document the natural variability in ice front position, ${\hat{Q}}_{r}^{\text{ref}}$, from its maximum position in winter versus summer (*5*, *6*), the rate of glacier retreat, *q*_r_, and the cumulative retreat, ${\hat{Q}}_{r}$, using time series of remote sensing imagery ($q_{r}^{\text{ref}}$ is 0). To balance mass fluxes at the ice front, we consider the rate of dry calving, *q*_c_, and its anomaly, ${\hat{Q}}_{c}$, from a reference $q_{c}^{\text{ref}}$ in 1992–1997. The cumulative anomaly, ${\hat{Q}}_{r}$, balances the sum of (${\hat{Q}}_{m}+{\hat{Q}}_{s}+{\hat{Q}}_{c}$) minus ${\hat{Q}}_{f}$.

### Glacier categorization

**Fig. 3:**
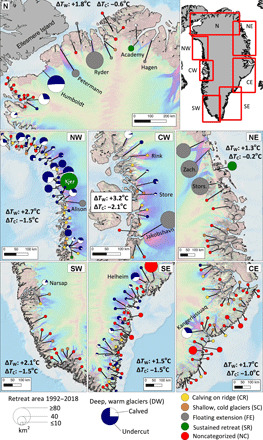
Spatial distribution of glacier retreat and categorization. Greenland glaciers (226) are classified by bathymetry and water properties as deep and warm (DW; blue/white circle), calving on a ridge (CR; yellow circle), shallow and cold (SC; brown circle), terminating in a FE (gray circle); and by those with sustained retreat (SR; green circle) or NC (red circle). Circles are proportional to the grounded ice loss in square kilometer for 1992–2017, with blue for undercutting versus other ablation processes on DW glaciers. Δ*T*_W_ and Δ*T*_C_ are net changes in ocean TF during the 1998–2007 and 2008–2017 periods, respectively. Background ice velocity map is from (*35*), and bathymetry is from (*19*).

To simplify the analysis of the relationship between glacier undercutting and the onset and progression of glacier retreat, we group the 226 glaciers into six categories. Four categories are based on the fjord/ice geometry and the detected presence of AW, pertaining to 135 glaciers that have sufficient measurements to characterize bathymetry and water properties: (i) 74 glaciers terminating in deep warm water (DW) with the detected presence of AW; (ii) 27 glaciers that break into icebergs on shallow ridges [calving ridges (CR)], independent of the presence of AW; (iii) 24 glaciers that stand in shallow cold (SC) (<100-m depth) fjords with polar water; and (iv) 10 glaciers with long (>10 km) floating extensions (FE). We partition the 91 remaining glaciers into two additional categories: (v) four glaciers already in an SR in 1992–1997 and (vi) 87 noncategorized (NC) glaciers due to a lack of bathymetry and ocean temperature data. The glacier distribution follows geography and precipitation regime (Fig. 3). DW glaciers dominate in NW, CW, and SE, where precipitation, glacier speed, and rates of mass turnover are high. SC glaciers are common in SW and CE, where mass turnover is lower. FE glaciers are in the cold, dry N and NE, except for Jakobshavn Isbræ and Alison. NC glaciers prevail in SW and CE where measurements of ice thickness and bathymetry are few, but the glaciers are thinner and the fjords are shallower than on average.

We find that the 74 DW glaciers (Fig. 2A) retreated 1228 km^2^ in 1992–2017, with a 30-fold increase from 1992–1997 (13.5 km^2^) to 1998–2017 (602 km^2^) (Table 1). In the cooling period 2008–2017, DW glaciers continued to retreat by 613 km^2^. During the 1992–2017 period, DW glaciers controlled 47% of the grounded ice loss and 49% of the total ice sheet mass loss (Table 1). In contrast, CR glaciers have been stable, contributing only 3% of the grounded ice loss and 9% of the total mass loss. CR glaciers terminate on ridges where they form temporary, short floating extensions that extend past the ridges before breaking into icebergs (Fig. 2B). In this configuration, undercutting by the ocean does not affect basal friction; it only melts floating ice, so CR glaciers are protected from warmer waters until undercutting increases enough to force a retreat past the ridge. CTDs available for 26 of 27 CR glaciers indicate the presence of AW within their fjords, and their stability is supported by model studies in NW Greenland (*30*). Similarly, SC glaciers (Fig. 2C) contributed only 3% of the grounded ice retreat and 5% of the total mass loss. Prior modeling of ice front melt suggested that, on shallow glaciers, melt rates peak in near-surface layers causing an “overcut” (*31*). While this process is not included in our study, the low rate of retreat and mass loss of SC glaciers suggest that it is not a significant destabilizing mechanism. In contrast, FE glaciers, which terminate in deeper fjords, contributed 22% of the grounded ice retreat, especially in 1998–2007 when several ice shelves disappeared. For Hagen and C. H. Ostenfeld, the ice shelf removal did not affect the flow speed; but for Jakobshavn, Zachariae, and Alison, the removal led to an acceleration in ice flow and grounded ice retreat. FE glaciers contributed 15% of the mass loss. The four SR glaciers continued to retreat the entire period but contributed only 5% of the retreat and 2.5% of the mass loss. For the 87 NC glaciers, the default bathymetry in BedMachineV3 is too shallow (e.g., Mælkevejen and Midgård) or ice thickness is too low (e.g., Heim, Sondre Parallel, Sermilik Bræ, and Fimbul) to constrain terminus geometry—a key component in estimating ocean-induced melt rates. However, NC glaciers only constituted 16% of the retreat and 19% of the mass loss (Table 1).

### Glaciers in deep fjords with warm AW

For glaciers in deep fjords with warm AW (DW), ice front undercutting and retreat increased when TF increased. During the stable 1992–1997 period, *q*_m_ averaged 0.69 m/day, and grounded ice loss averaged 2 km^2^/year (Table 2). In the 1998–2007 period, *q*_m_ increased by 48% to 1.02 m/day, and grounded loss increased to 60 km^2^/year. The increase in undercutting varied regionally, being lower in southern sections, where TF was initially higher: *q*_m_ increased 31% in SE, where TF initially averaged 6°C but by 75% in NW, where TF initially averaged 2.5°C. As TF declined in 2008–2017, undercutting decreased by 1% and hence remained higher than in 1992–1997, and ice fronts continued to retreat at 61 km^2^/year.

**Table 2:** Summary of glaciers in deep fjords with warm AW. Grounded ice loss in square kilometers and mean undercutting *q*_m_ in meters per day, for DW glaciers during three time periods; and cumulative undercutting, ${\hat{Q}}_{m}$, cumulative dry calving, ${\hat{Q}}_{c}$, and thinning-induced retreat, ${\hat{Q}}_{s}$, as a fraction of the total mass ablation at the ice front = $\left({\hat{Q}}_{m}+{\hat{Q}}_{c}+{\hat{Q}}_{s}\right)$ for the entire 1992–2017 period.

**Coastal sector**	**No. of DW** **glaciers**	**Ice loss (km^2^)**			***q*_m_ (m/day)**			**Percentages of total ablation**		
		**1992–1997**	**1998–2007**	**2008–2017**	**1992–1997**	**1998–2007**	**2008–2017**	**${\hat{Q}}_{m}$**	**${\hat{Q}}_{c}$**	**${\hat{Q}}_{s}$**
CW	6	−2.4	32.0	53.3	0.58	0.89	0.97	30.7	68.5	0.9
SW	1	−0.1	2.8	18.2	0.79	0.81	1.01	4.3	95.7	0.0
SE	24	15.3	103.6	74.6	1.25	1.64	1.54	58.5	31.0	10.5
CE	2	−0.6	24.6	17.0	0.33	0.57	0.92	38.7	9.1	52.3
NE	0	–	–	–	–	–	–	–	–	–
N	6	17.9	163.3	177.9	0.06	0.23	0.26	48.5	39.0	12.5
NW	35	−16.6	274.7	272.9	0.44	0.77	0.79	75.0	8.7	16.3
Total	74	13.5	601.9	612.9	0.69	1.02	1.01	62.0	25.6	12.4

**Fig. 4:**
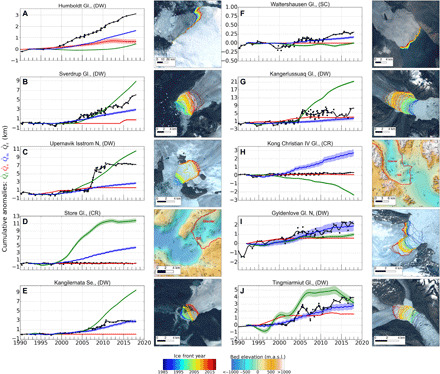
Summary of cumulative anomalies at the glacier terminus for ten examples. (**A** to **J**) Example glaciers for the seven regions showing time series of observed ice front retreat, ${\hat{Q}}_{r}$ (black), retreat induced by surface thinning, ${\hat{Q}}_{s}$ (red), and cumulative anomalies in undercutting by the ocean ${\hat{Q}}_{m}$ (blue) and in ice advection, ${\hat{Q}}_{f}$ (green), for 1992–2017. Second and fourth columns show the ice front location color coded from 1992–2017 overlaid on Landsat 8 imagery or bed elevation. Note that the vertical axis is scaled differently for each example.

We find a broad agreement between the timing of enhanced undercutting above the range of natural variability of the calving front position, ${\hat{Q}}_{r}^{\text{ref}}$, and the initiation of retreat for DW glaciers for almost all glaciers (Fig. 4). For example, at Humboldt (N), the ice front retreat in year 2000 coincides with a positive ${\hat{Q}}_{m}$ (Fig. 4A). The sum of ${\hat{Q}}_{m}$ and ${\hat{Q}}_{s}$ matches the observed retreat, ${\hat{Q}}_{r}$, well. A comparable relationship between undercutting and retreat is found for Sverdrup and the northern branch of Upernavik Isstrøm in NW (Fig. 2, B and C), Kangerlussuaq in CE (Fig. 4G), and Tingmiarmiut in SE (Fig. 4J). At Kangilernata in CW (Fig. 4E) and the northern branch of Gyldenlove in SE (Fig. 4I), we find that ${\hat{Q}}_{m}$ replicates ${\hat{Q}}_{r}$ almost entirely while ${\hat{Q}}_{s}$ and ${\hat{Q}}_{c}$ did not change, which provides confidence in the modeling of undercutting. On average, we calculate that the retreat of DW glaciers follows the increase in undercutting by 0.8 years. This delay corresponds to a forced retreat of 100 m for an increase in *q*_m_ of 0.3 m/day, i.e., comparable to the natural variability of the ice front, ${\hat{Q}}_{r}^{\text{ref}}$. We attribute delays between ${\hat{Q}}_{m}$ and ${\hat{Q}}_{r}$ to variations in ${\hat{Q}}_{r}^{\text{ref}}$ from one glacier to the next and uncertainties in the parameterization of *q*_m_.

On DW glaciers, ${\hat{Q}}_{m}$ controlled 62% of the mass removal, i.e., (${\hat{Q}}_{m}+{\hat{Q}}_{c}+{\hat{Q}}_{s}$), versus 12% for thinning-induced retreat ${\hat{Q}}_{s}$ and 26% for dry calving ${\hat{Q}}_{c}$ (Table 2). We conclude that the retreat at DW glaciers is driven by undercutting. Undercutting dominates in SE and NW, i.e., high-precipitation regions, whereas dry calving dominates in CW and N. Thinning-induced retreat is not a major mode of retreat—48 of 74 DW glaciers retreated as a result of surface thinning with an average ${\hat{Q}}_{s}$ of 1.2 km between 1992 and 2017—except for a few notorious glaciers that include Helheim (SE) where ${\hat{Q}}_{s}$ is 4.4 km and Kangerlussuaq (CE) with a ${\hat{Q}}_{s}$ of 3.1 km.

For glaciers calving off a ridge at the terminus (CR), ${\hat{Q}}_{m}$ did not affect the ice fronts because the glaciers, e.g., Store in CW (Fig. 4D) and Kong Christian IV in CE (Fig. 4H), calve on a ridge with small, temporary, floating sections. CR glaciers are dominated by calving processes unless *q*_m_ increases considerably more than for DW glaciers to dislodge the glaciers from the ridges. For shallow glaciers in contact with polar water (SC), we estimate minor changes in *q*_m_, consistent with a lack of retreat. For Waltershausen in NE, ${\hat{Q}}_{m}$ is less than 1 km in 1992–2017 versus a minimal retreat (Fig. 4F). For FE glaciers, most of the retreat corresponded with ice shelf collapse. For NC glaciers, the uncertainties are too large to determine the role of the ocean, which illustrates the importance of measuring bathymetry and ocean temperature in all the fjords.

## DISCUSSION

This study demonstrates that a large, abrupt ∼1.9°C warming of subsurface ocean waters around Greenland in 1998–2007 relative to 1992–1997 triggered a widespread mass removal at the front of marine-terminating glaciers, which forced them into an state of retreat. Cumulative anomalies in undercutting of 74 major glacier fronts standing in deep warm fjords increased mass removal at the ice front. For these glaciers, 62% of anomalous grounded ice removal was caused by ocean-induced undercutting, while the remainder was split between thinning-induced retreat (12%) and anomalous dry calving (26%). DW glaciers contributed 61% of Greenland’s loss from ice dynamics and 49% of the total loss. FE glaciers contributed 19% of the loss from ice dynamics and 15% of the total loss. NC glaciers contributed 10 and 20%, respectively. Many of the NC glaciers have likely been affected by ocean warming because their retreat rate increased 10-fold between the stable and warming periods, but we lack the oceanographic data required to quantify this relationship. The remaining glaciers (CR, SC, and SR) contributed 11% of the loss from ice dynamics and 17% of the total mass loss. DW glaciers therefore played a dominant role in the changes in ice dynamics in Greenland and, in turn, on the total ice sheet mass loss. Their retreat broadly coincided with the warming of subsurface ocean waters in location, time, and magnitude of undercutting.

As ocean warming paused in 2008–2017, the net ice discharge from Greenland glaciers kept increasing, ice fronts kept retreating, and the rate of undercutting remained higher than in the previous decade. In effect, the changes in ocean conditions that took place in the mid-1990s did not revert to their initial state and had a profound influence on the stability and evolution of Greenland glaciers. We posit that the sustained dynamic thinning at glacier margins, particularly after 2000 (table S1), led to an unstable configuration of the glaciers, which could not reach a new equilibrium state.

In this study, we document changes in the calving rates associated with changes in ice advection rates near the terminus by deducing them from the mass balance equation. We cannot identify the particular mechanisms that lead to dry calving, e.g., extensive longitudinal stretching or rotation of grounded ice blocks near floatation; hence, care must be exercised when comparing our dry calving rates to those from other studies. We also do not have an independent method to determine the uncertainty in dry calving or undercutting. If we consider glaciers for which the cumulative anomaly in ice advection ${\hat{Q}}_{f}$ was small (<1 km) over 1992–2018, then we find that ice front retreat is well explained by the combination of thinning-induced retreat and the cumulative undercutting anomaly, i.e., ${\hat{Q}}_{r}\approx {\hat{Q}}_{s}+{\hat{Q}}_{m}$, which validates a posteriori our component estimates ${\hat{Q}}_{m}$ and ${\hat{Q}}_{s}$. For example, at Waltershausen Glacier (Gl.) and Gyldenlove N (Fig. 4, F and I, respectively), we find ${\hat{Q}}_{s}=0$ and ${\hat{Q}}_{m}\approx {\hat{Q}}_{r}$. While more progress is desirable to gain confidence in the modeled rates of undercutting, the ice sheet–wide results already provide a realistic match with 26 years of observations of all glaciers with quality bathymetry and ocean temperature data.

Our results have implications for the projection of ice sheet mass loss with oceanic and atmospheric forcing scenarios. First, when glaciers are perturbed at their terminus by increased undercutting rates, the glaciers speed up and thin, which is conducive to more retreat if the ice fronts are close to floatation and not calving on a ridge. This feedback cannot be captured by a linear approximation of retreat based on regional ocean forcing (*17*) as prescribed in the most recent Ice Sheet Model Intercomparison Project (ISMIP6, *32*). For example, glaciers continued to retreat in SE Greenland after 2010, even as TF values returned to lower values (Fig. 1). Second, model projections used to forecast sea-level rise from the Greenland Ice Sheet do not include realistic ice-ocean interactions because they lack the novel bathymetric and ocean TF details required to reproduce the rates of undercutting that force the retreat. If these models do not incorporate a forced retreat by undercutting, then they will produce retreat rates that are too low compared to observations. In the absence of realistic ocean TF and ensuing feedbacks, ice sheet numerical models will therefore underestimate the mass loss of Greenland glaciers. As warmer waters controlled more than half of the mass removal at calving margins in our ice sheet wide analysis and ocean TF is expected to increase in the coming decades (*33*), current numerical models may underestimate future mass losses by at least a factor of 2.

## MATERIALS AND METHODS

### Ice front retreat

Ice front retreat is manually digitized from level 1 Landsat 5, 7, and 8 products from 1985 to 2018. The resolution of Landsat data is 30 m for Landsat 5 (1985–1999) and 15 m for Landsat 7 and 8 (1999–2013 and 2013–2018, respectively). Ice front positions digitized as part of this project were supplemented with those publicly available from (*6*, *7*, *34*). We formulate an areal record of ice front retreat by calculating the total change in area between successive fronts and within the fjord boundaries. We calculate a linear record of ice front retreat by dividing the areal record by the mean width of the glacier front in between two epochs. This procedure yields results similar to the “box method” (*6*), where the width of the box is determined by the average width of the glacier front. We apply a 1-year smoothing to the linear record of retreat. We estimate the natural range of seasonal variability in ice front position (${\hat{Q}}_{r}^{\text{ref}}$) by comparing the maximal (end of winter) and minimal (end of summer) glacier extents between 1985 and 1992 where sufficient (≥5 years of) data are available, i.e., before the main retreat. Where we lack sufficient data, we calculate ${\hat{Q}}_{r}^{\text{ref}}$ from the SD between glacier length and the 1-year smoothed record using ice front data during years for which more than two observations are available.

### Ice advection

Ice advection, *q_f_*, is extracted from satellite radar and optical data (*1*, *35*). Observations are averaged over 1-year period from 1 July to 30 June, as most radar-derived velocity data were acquired in winter. Annual speed is averaged on a line parallel to the glacier terminus and positioned 1 km upstream to minimize data noise.

### Ice front undercutting

Ice front undercutting, *q*_m_, is derived from a series of high-resolution [*O*(1 m)] simulations using the MITgcm ocean model (*20*, *21*) with varying water depth, subglacial water runoff production, and ocean TF. On the basis of the ensemble simulations, modeled undercutting is parameterized as $q_{m}=(A\;b\;q_{\text{sg}}^{\alpha }+B)\;TF^{\beta }$, where *b* is the water depth, *q*_sg_ is the subglacial discharge averaged over the glacier front area; TF is the depth-averaged ocean TF in the lower 60% of the water column; and *A*, α, *B*, and β are constants (*20*). All variables (*b*, *q*_sg_, and TF) are averaged across the submerged calving face and updated monthly, yielding a monthly record of undercutting in meters per day. After selection of a reference undercutting value, $q_{m}^{\text{ref}}$, we construct the cumulative anomalies in undercutting, *Q*_m_, in meters.

The uncertainty in *q*_m_ combines uncertainties in water depth, *b* (10 m); subglacial discharge, *q*_sg_ (20%); and TF (0.3°C), with a 15% uncertainty due to the unknown spatial distribution of subglacial discharge as calculated in (*21*) and a 7% uncertainty associated with model fitting as discussed in (*20*). The mean nominal uncertainty in *q*_m_ is 26%. At the regional level, the uncertainty is probably larger than 26% but difficult to estimate. On Store Glacier, we calculate a mean rate of melt of 2.0 ± 0.3 m/day versus an observed rate of 3 ± 1 m/day (*21*). Observations of undercutting in Alaska (*23*) reveal undercutting rates of 8.5 m/day in August and 1 m/day in winter on a shallow glacier with 200-m water depth [Fig. S6 in (*23*)]. We tested whether our method reproduces these high Alaska melt rates using the following data from (*23*): a TF of 9°C in August and 6°C in May; a *q*_sg_ of 270 m^3^/s from table S2 therein; a cross-sectional area of 150,000 m^2^ from Table 1 therein; and an average depth of 170 m. We calculate a *q*_m_ of 7 m/day in August and 3 m/day in May, which are reasonably close to observations. The largest residual uncertainty is associated with the transfer coefficient for salt and heat (*24*).

#### Water depth

Water depth, *b*, is from BedMachineV3 (*19*), which uses multibeam echo sounding bathymetry in the fjords (±2 m), supplemented with bathymetry inferred from airborne gravity data (±60 m) in NW, CW, and SE (*36*, *37*). Water depth has an uncertainty of less than 10 m.

#### Subglacial discharge

Subglacial discharge, *q*_sg_, combines basin-integrated runoff from the University of Utrecht’s Regional Atmospheric Climate Model (RACMO2.3p2) at 5.5-km spatial resolution, statistically downscaled to 1 km (*38*) combined with basal melt water production beneath grounded ice calculated by the Ice Sheet System Model (ISSM) (*39*). The uncertainty in *q*_sg_ is 20%.

#### Thermal forcing

TF is the depth-averaged difference between the in situ temperature and the salt- and pressure-dependent freezing point of seawater. Ocean state parameters are sampled from two ECCO ocean state estimates: (i) a high-resolution (4 km) forward model in an Arctic domain run from an initial state for the period 1992–2011 (*3*) and (ii) a data-constrained global estimate with 13.5-km horizontal resolution around Greenland for the period 2001–2017 (LLC270) (*40*). Each product is sampled and horizontally averaged in 28 calibration areas around Greenland (Fig. 1). The 4-km product, calibrated and evaluated using CTD data, has sufficient spatial resolution to resolve the transfer of ocean heat from the outer ocean onto the continental shelf. The 4-km solution is adjusted for two biases: (i) a +0.4°C overall bias in all sample areas at depths below 50 m and (ii) an initial −1°C bias in Baffin Bay, which decreased through time as the model became closer to observations. For the bias in Baffin Bay, a linearly varying correction is applied to NW1 to NW4 and CW1 to CW2 for depths below 50 m using (2012 − *t*)/20, which is +1 at the start of the simulation in 1992 and 0 at the end of the simulation in 2012. The mean adjustment to the 4-km solution, δ_4km_, is listed in table S1. The LLC270 product, derived to match regional oceanographic observations, does not have sufficient spatial resolution to reproduce off-the-shelf to on-the-shelf ocean heat transport processes. To correct for biases in the LLC270 solution, a depth-dependent mean difference profile is deduced from a comparison with the 4-km solution in each calibration area during the overlapping period 2001–2011. The LLC270 solution is adjusted with this mean profile for the entire series 2001–2017. The mean adjustment for the LLC270 is listed in table S1. The horizontally averaged model solutions are merged using a linear weighting over 2009–2011 to create a seamless TF series. The merged time series are then adjusted with an absolute shift applied to the entire time series to best match the CTD data from the OMG campaign (see Data and materials availability) and other sources (*41*) within the calibration area. These final adjustments and root mean square errors are listed by calibration area in table S1. The root mean square error between the reconstructed TF and CTD data is 0.55°C. Because the spatial resolution of the ECCO state estimates is not sufficient to resolve heat transport into glacier fjords, the merged and adjusted time series from the closest 28 calibration areas are further adjusted to best match the historical CTD data within the fjords, primarily available from the OMG campaign (see Data and materials availability). For each glacier, the TF reconstruction (table S1) is integrated over the deepest 60% of the water column in front of the glaciers across the geometry of the glacier terminus. We estimate the nominal precision in calibrated TF to be 0.3°C.

### Thinning-induced glacier retreat

Thinning-induced glacier retreat, *q*_s_, is calculated using a simple, geometrically derived relationship for grounding line migration rate as a function of surface elevation change: *q*_s_ = *dh*/*dt*/[(1 − ρ_w_/ρ_i_)β_s_ − α_s_] (*42*), where *q*_s_ is positive for retreat, *dh*/*dt* is the surface thinning rate (thinning is positive), ρ_w_ is the density of seawater, ρ_i_ is the density of ice, β_s_ is the basal slope at the ice front (or grounding line for ice shelves), and α_s_ is the slope of the glacier surface at the ice front (or grounding line for ice shelves). We apply this formula using DEMs from three time periods: (i) 1978–1987 (*27*), (ii) 2002–2009 (*28*), and (iii) 2015–2016 (*29*) interpolated in between using time series of laser altimetry data from Operation Ice Bridge and time series of ice velocity as the basis for interpolation as in (*1*). Results are summarized by regions and glaciers in table S1.

Surface and basal slopes, α_s_ and β_s_, are measured over a distance of 10 ice thicknesses behind the grounding line and are positive when the bed elevation or surface elevation increases seaward. α_s_ is calculated from a time series of ice surface elevation (*1*). β_s_ is calculated from BedMachineV3. Ice elevation change, *dh*/*dt*, is obtained by differencing the time series of ice surface elevation on 1-km segments centered on and perpendicular to the center flow line of the glacier, sampled every 100 m, and truncated on the basis of the time-dependent position of the ice front. Height above floatation is the difference between the ice surface elevation, *h*, and the floatation height, *h*_f_ = *b*(ρ_w_ − ρ_i_)/ρ_i_, where ρ_w_ is the density of seawater and ρ_i_ is the density of ice. Thinning-induced retreat distance is calculated over the time period after which the glacier thinned to floatation, δ*t*_s_, and multiplied by the thinning-induced retreat rate, *q*_s_, i.e., ${\hat{Q}}_{s}=q_{s}\delta t_{s}$. When the glacier does not thin (*dh*/*dt* ≥ 0), we use δ*t*_s_ = 0.

### Mass conservation at the ice front

Mass conservation at the ice front dictates that *q*_r_ = *q*_m_ + *q*_s_ + *q*_c_ − *q*_f_. We measure *q*_r_ and *q*_f_, calculate *q*_m_ and *q*_s_, and deduce *q*_c_ from the mass balance equation. When integrated over the entire period into cumulative anomalies, we verify that ${\hat{Q}}_{r}$ = ${\hat{Q}}_{m}$ + ${\hat{Q}}_{s}$ + ${\hat{Q}}_{c}$ − ${\hat{Q}}_{f}$, with a reference state of zero for *q*_r_ and *q*_s_ (no retreat and no thinning in a state of equilibrium) and reference states for *q*_m_, *q*_c_, and *q*_f_ based on the reference time period 1992–1997 when most glaciers were close to a state of mass balance (see table S1).

## Supplementary Material

http://advances.sciencemag.org/cgi/content/full/7/1/eaba7282/DC1

Table S1

Adobe PDF - aba7282_SM.pdf

Ocean forcing drives glacier retreat in Greenland

## SUPPLEMENTARY MATERIALS

Supplementary material for this article is available at http://advances.sciencemag.org/cgi/content/full/7/1/eaba7282/DC1
