# Supplementary material for: Ocean forcing drives glacier retreat in Greenland
Source: Sci Adv. 2021 Jan 1;7(1):eaba7282. doi: 10.1126/sciadv.aba7282 (PMC7775757; doi:10.1126/sciadv.aba7282)
Supplement: http://advances.sciencemag.org/cgi/content/full/7/1/eaba7282/DC1 [file supp_7_1_eaba7282__index.html]

Science Advances | Science AdvancesAAASSearchScience AdvancesMenu

## Supplementary Materials

# Ocean forcing drives glacier retreat in Greenland

Michael Wood, Eric Rignot, Ian Fenty, Lu An, Anders Bjørk, Michiel van den Broeke, Cilan Cai, Emily Kane, Dimitris Menemenlis, Romain Millan, Mathieu Morlighem, Jeremie Mouginot, Brice Noël, Bernd Scheuchl, Isabella Velicogna, Josh K. Willis, Hong Zhang

Download Supplement

**Other Supplementary Material for this manuscript includes the following:**

- Table S1

**Files in this Data Supplement:**

- Adobe PDF - aba7282\_SM.pdf
- aba7282\_Table\_S1.xlsx
