# Supplementary material for: Ocean forcing drives glacier retreat in Greenland
Source: Sci Adv. 2021 Jan 1;7(1):eaba7282. doi: 10.1126/sciadv.aba7282 (PMC7775757; doi:10.1126/sciadv.aba7282)
Supplement: http://advances.sciencemag.org/cgi/content/full/7/1/eaba7282/DC1 [file supp_7_1_eaba7282__1.pdf]

Science Advances
AAAS

advances.sciencemag.org/cgi/content/full/7/1/eaba7282/DC1

Supplementary Materials for

Ocean forcing drives glacier retreat in Greenland

Michael Wood*, Eric Rignot, Ian Fenty, Lu An, Anders Bjørk, Michiel van den Broeke, Cilan Cai, Emily Kane, Dimitris Menemenlis, Romain Millan, Mathieu Morlighem, Jeremie Mouginot, Brice Noël, Bernd Scheuchl, Isabella Velicogna, Josh K. Willis, Hong Zhang

*Corresponding author. Email: mhwood@uci.edu

Published 1 January 2021, *Sci. Adv.* **7**, eaba7282 (2021)
DOI: 10.1126/sciadv.aba7282

Other Supplementary Material for this manuscript includes the following:

(available at advances.sciencemag.org/cgi/content/full/7/1/eaba7282/DC1)

Table S1
